# The Life and Work of Charcot

**Published:** 1894-05

**Authors:** William C. Krauss

**Affiliations:** 382 Virginia Street; Buffalo, N. Y.


### THE LIFE AND WORK OF CHARCOT.

By WILLIAM C. KRAUSS, M. D., Buffalo, N. Y.

On the 16th of August, 1893, there flashed over the wires from the Lac des Settons the report of the sudden death of Jean Martin Charcot. Seldom has the death of any scientific man caused such universal grief, not alone in France, but throughout the civilized world wherever the medical sciences are taught and recognized. The public as well as the medical press, in reporting the facts of his life and work, spoke in but one tone of the magnitude of his labors and of the irreparable loss to the profession.

Jean Martin Charcot was born in the City of Paris, of humble but honest parents, November 29, 1825. His father, too poor to give his three sons a collegiate education, summoned them to his side one day and announced that the one who would stand highest in his year's work could continue his education, while the second *could* be a soldier and the third *would* have to become a coach-maker, like himself. The contest over, "Charcot" was sent to Lycée St. Louis, then to the University of Paris, and in 1848 was received as *interne* to the hospitals. Five years later he passed his doctor examinations and wrote his thesis on nodosities of the joints, chronic articular rheumatism. This first production of Charcot showed him to be a careful, painstaking worker and observer, and as a result he was made chef of the medical clinic from 1853 to 1855. At the end of his term of service he was appointed physician to the Bureau Central, and four years later passed his examinations as Agregé of the medical faculty of Paris. This position is similar to the Privat Docent of the German universities, and may be compared to the lecturers on special subjects of our American medical colleges. His thesis as Agregé, on chronic pneumonia, has been often referred to and quoted. In this thesis he describes that form of pneumonia which is designated chronic ulcerated pneumonia.

In 1862, Charcot returned to the Salpetriêre as chef of the service. At this time the Salpetriêre was nothing but an out-of-the-way asylum for old, chronic, broken-down cases, patients who were thought not to be worth the time and trouble to thoroughly examine and scrutinize. It was not long, however, before Charcot, associated with his able friend, Vulpian, discovered that these chronic castaways were diamonds in the rough, and needed but a master hand, guided by a master mind, to reveal their significance and utility to the scientific world. The old stagers were carefully examined, assorted, their clinical histories thoroughly written out, abnormalities and disturbances of motion photographed, chemical and microscopical examinations of the excreta were made from time to time, and the information thus gained was pigeonholed, ready for additions and for perusal at the autopsies. These were made with as much care and precision as are many of our major operations of today, and the diseased organs were subjected to a most conscientious microscopical examination. Not only were the pathological changes noted, but to a greater extent were those organs which had not undergone pathological change. Thus armed with the symptoms of disease and the diseased and healthy organs and tissues under the microscope, it was an easy task for this man to clear up and efface many of the illusions of medicine which had existed up to that time.

After an inventory of years, in which time the Salpetriêre had become one of the best known of the Paris hospitals, Charcot believed that he could better impart the information thus gained by inaugurating a course of lectures and presenting the patients before the class. These were especially devoted to pneumonia of the aged, chronic rheumatism, and gout, and were attended by an appreciative and admiring throng of students. The following year he delivered his lectures at the École Pratique, on Hemorrhage and Softening of the Brain, and made the important discovery that cerebral hemorrhage was due to the bursting of miliary aneurisms along the cerebral arteries, the result of a periarteritis. The year 1858 was devoted to the study of paralysis agitans and sclerose en plaque (multiple sclerosis). Many of the disputed points of the former disease were cleared away, besides making new additions to the symptomatology, such as propulsion, retropulsion, the characteristic tremulous movements of the extremities, absolute freedom of the head, except when the movement is transmitted from the body and extremities. Charcot has objected strenuously to the term paralysis agitans, inasmuch as the tremor may be absent altogether and the only symptoms present be the stiffness and weakness of the muscles. Multiple sclerosis, or the presence of scattered islets of sclerosis in the brain and spinal cord, was illy known and almost unrecognized until the classical descriptions of the symptomatology and pathology by Charcot, assisted by Vulpian. Charcot distinguished three forms of this disease, the cerebral, the spinal and the cerebro-spinal, according to the situation of the sclerosed patches. Little has been added to the description as given by Charcot, and, perhaps, the only symptom that has not been universally accepted in that description is the severe vertigo which Charcot found in seventy-five per cent. of his cases.

In his course of lectures delivered in 1869, Charcot treated of the pathogenesis, the diagnosis and prognosis of cerebral apoplexy. These lectures were not reported, although they contained many new points which have since been incorporated in theses by internes of the Salpetrière. In this year, Charcot called attention to the importance of thermometry in the diseases of old age, and it is principally to his efforts that the clinical thermometer was vulgarized in France. It was also in this year that Charcot first described the peculiar joint affections occurring in locomotor ataxia; the affected bones become brittle, resulting in spontaneous fractures, erosion of the cartilages, adhesions between two joint surfaces, ossification of ligaments, etc., take place. This condition is known as Charcot's joint disease, a term applied to it by the English writers. Charcot was a bitter opponent to the Fournier-

Erb doctrine, that tabes is of syphilitic origin, but taught that it belonged to the great family of nervous diseases, the seeds of which are transmitted by heredity. It may be remarked here that the Argyl-Robertson pupil (refusal of the pupil of the eye to react to light and darkness) was first discovered at the Salpetriêre by two of Charcot's pupils, Vincent and Congt. Disputed points in the pathology and advances in treatment were also ably considered, and especially the re-introduction of the suspension method, which gives, perhaps, as much satisfaction and relief as any method of treatment in locomotor ataxia. In 1869, Charcot, associated with Vulpian and Brown-Sèquard, founded the *Archives de Physiologie*, the ablest and most influential medical journal in France, and with the possible exception of *Virchow's Archives*—the ablest on the continent. During the Franco-German war Charcot, besides his regular duties at the Salpetriêre, assumed charge of a small-pox hospital and hospital barracks. It may be noted here that he was the court physician of Napoleon III. for many years, and in sympathy ever afterwards with the royalists. The service at the Salpetriêre was divided into three divisions after the war, owing to the vast increase in numbers, and to Charcot was assigned the hysterical and epileptics. In 1872, he was appointed professor of pathology in the University of Paris, a position which he held until 1881. During this time he published his observations on diseases of the lungs, liver and kidneys, albuminuria, diseases of old age, and on cerebral and spinal localization. His discovery of the asthma crystals, the presence of octahedral crystals in the blood, spleen, bone marrow, and other organs of leukemic patients, his contributions to the pathology of hepatitis, cancer of the lungs, gout, arthritis deformans, etc., show him to have been as good an observer in general pathology as in neuropathology. In 1882, the chair of nervous diseases was created in the faculty of the University of Paris, and Charcot had the honor of being appointed professor. His attention was directed now entirely to his chosen work, and as a result we have the creation of the modern malady hysteria, its symptomatology, different stages, different forms, its historical importance, modes of treatment; in short, about all we know positively concerning hysteria we owe to Charcot and his pupils. To enumerate all the advances which he instituted in neuropathology, would require more time, more paper, and more ability, than are at my command—in fact, it would mean an almost complete treatise on neurology. I will, therefore, review briefly the more important of his discoveries, which will give you some idea of the grandeur of his life's work. In 1871, he first described pachymeningitis cervicalis hypertrophica, which was at once accepted as a clinical and pathological entity. In 1874, he described a new disease which he designated amyotrophic lateral sclerosis, sometimes called Charcot's disease, with such clearness as to symptoms, course and pathology that the German and English writers adopted the name and disease without even the slightest protest. So thoroughly were these two last affections delineated that scarcely a word has been added to the original descriptions. In 1875, Charcot and Erb described simultaneously an affection—met with particularly in children—characterized by paresis of the extremities associated with exaggeration of the tendon reflexes. Charcot designated this disease tabes dorsalis spasmodique, Erb, spastic spinal paralysis. Infantile spinal paralysis was first described as a disease by Jac. von Heine in 1840, but it remained for Charcot, in 1873, to discover the pathological lesion in the anterior horns, and led him to promulgate the most important law in spinal pathology—namely, that destruction of the lateral group of ganglion cells in the anterior cornua is followed by atrophy of the muscles and loss of tendon reflexes. In 1870, Charcot found that in those patients suffering with glosso-labio-laryngeal paralysis (bulbar paralysis) there was a progressive destruction of the ganglion cells in the medulla and pons presiding over these parts. Leyden, of Berlin, soon thereafter made the same discovery, independent of Charcot.

The discovery of the lesion in the Duchenne-Aran type of progressive muscular atrophy in the anterior cornua was due to Charcot. Stubbornly opposed for a time was the German school, headed by Friedreich, who believed that the atrophy was due to a myositis, and the nerve and spinal cord changes were considered as sequelæ. Charcot's theory is now universally accepted. In 1886, he, associated with Marie, described the peroneal or leg-type of progressive muscular atrophy, at about the same time Tooth, of London, made the same discovery. Much that we know of the pathology of myelitis, and compression myelitis, syringomyelia, Morvan's disease—in short, there is not an organic lesion of the spinal cord which has not the name Charcot stamped upon it.

In cerebro-pathology we owe to him, besides the discovery of the miliary aneurisms in cerebral apoplexy, the location of the agraphic center, the location of the lesion in athetosis, the path of fibers in the internal capsule and the discovery of the carrefour sensitiv. He has given valuable hints in regard to epilepsy, chorea, and post-hemiplegic chorea, astasia-abasia, the urinary paraplegias, the traumatic neuroses, male hysteria and sciatica.

It has been said that Charcot did not pay as much attention to therapeutics as could have been expected of one with such boundless opportunities. This criticism I believe unjust. In 1876, he revised the work of Burq on metalloscopie and metallotherapie, introducing the magnet as a therapeutic agent, and other discoveries in the treatment of the hysterical, notably the value of ovarian pressure in aborting attacks, and all that is scientific and uncharlatan in the use of hypnotism as a therapeutic agent. His views on the latter are so firm and so clear regarding the proper use of hypnotism, its nature and powers, that they have received the appellation—Charcot's doctrine. He first advised the quinine treatment in Menière's disease, the use of the cautery in paraplegia, the syphilitic treatment of Jacksonian epilepsy, which, by the way, was first discovered at the Salpetrière.

Charcot was elected a member of the Academy of Medicine of Paris in 1872, a member of the Institute of France in 1883, commander of the Legion of Honor in 1892, besides receiving orders and decorations from many of the crowned heads of Europe. Besides founding the *Archives of Physiologie*, he aided Bourneville in founding the *Lè Progrès Medical* in 1876, and in 1880 founded the *Archives of Neurologie.* He also created the *Revue Mensuelle de Medicine et de Chirurgie* in 1873, and was instrumental in calling to life the *Iconographie Nouvelle de la Salpetriére.*

The published works of Charcot consist of five volumes on nervous diseases, five volumes on diseases of the liver, lungs, kidneys, diseases of old age and infectious diseases, two volumes of the Leçons de Mardi, and two volumes of Charcot's clinic, besides innumerable essays—partly alone, partly in collaboration with his internes—on special subjects in general and neuropathology. Among those who comprise the school of the Salpetrière (his former internes and followers) we find such names as Joffroy, Fèrè, Bourneville, Cornil, Gombault, Marie, Babinski, Guinon, Pierret, Bloch, Brissaud, Huet, Gilles de la Tourette, Colin, Richer, Pitres, names of men all of whom are well known in medicine. Charcot was not only a physician but an artist of some celebrity, a thorough musician, and a lover of things beautiful.

As a man in the broad sense of the word he was peerless—kind, obliging, considerate and generous to the lowest as well as the highest in society's scale, one who shunned the glare of fraud and hypocrisy in his profession, he bequeathed to his science more than any man in France, if not in Europe, and leaves a monument to his memory in the heart and mind of everyone who ever had the pleasure of meeting with him in the Salle des Cours of the Salpetriêre.

382 Virginia Street.

